# Effectiveness of fermentation broth of *Cordyceps sinensis* for primary insomnia: a randomized clinical trial with digital health tool

**DOI:** 10.3389/fneur.2025.1555010

**Published:** 2025-07-11

**Authors:** Shuting Zhao, Ziqian Wang, Xiaxia Fan, Xuanhao Shu, Qiao Chen, Yibo Zhou, Yongzhi Fu, Zheng Yu, Marcin Grzegorzek, Xinyu Huang, Chengwen Zheng, Yanxiong Gan, Chuanbiao Wen

**Affiliations:** ^1^School of Intelligent Medicine, Chengdu University of Traditional Chinese Medicine, Chengdu, Sichuan, China; ^2^School of Acupuncture and Tuina, Chengdu University of Traditional Chinese Medicine, Chengdu, Sichuan, China; ^3^Key Laboratory of Acupuncture for Senile Disease (Chengdu University of TCM), Ministry of Education, Chengdu, Sichuan, China; ^4^Chengdu Zen Healing TCM Clinic, Ltd., Chengdu, Sichuan, China; ^5^Institute for Medical Informatics, University of Lübeck, Luebeck, Germany; ^6^German Research Center for Artificial Intelligence, (DFKI), Lübeck, Germany; ^7^Sichuan Provincial Engineering Technology Research Center for Digitalization of Traditional Chinese Medicine, Chengdu, Sichuan, China

**Keywords:** primary insomnia, *Cordyceps sinensis*, fermentation broth, digital health tool, Pittsburgh sleep quality index

## Abstract

**Background:**

*Cordyceps sinensis* is widely used in Traditional Chinese Medicine and dietary supplements to tonify the kidney, lung, and heart, as well as to calm the mind. The fermentation broth of *Cordyceps sinensis* (FBCS), containing cordycepin, has shown potential in various healthcare applications.

**Methods:**

Ninety patients with primary insomnia were divided into two groups: the FBCS group (*n* = 45) and the control group (*n* = 45). The FBCS group received Cordyceps fermentation liquid drink (150 ml/day), while the control group received a placebo (150 ml/day). Both groups were supported by Digital Health Tools (DHT) for medication supervision. Pittsburgh Sleep Quality Index (PSQI) scores were assessed and collected through the DHT application at the initial baseline period, 14, and 28 days after treatment.

**Results:**

Significant main effects of time and time × group interaction on the PSQI total score and all subcomponents. Significant group effects were also observed for most subcomponents, except for Sleep Disturbances and Daytime Function, indicating greater improvements in the FBCS group over time. After 14 and 28 days of FBCS consumption, the treatment group demonstrated greater reductions in PSQI total scores compared to the control group. Subcomponent analysis revealed greater improvements in the FBCS group compared to the control group at both time points. Specifically, Sleep Quality scores decreased by −0.89 vs. −0.50 at Day 14 and −1.32 vs. −0.45 at Day 28. Sleep Onset Latency improved by −0.75 vs. −0.20 at Day 14 and −1.32 vs. −0.27 at Day 28. Sleep Duration scores decreased by −0.86 vs. −0.20 at Day 14 and −1.20 vs. −0.23 at Day 28. Sleep Efficiency showed a decline of −0.82 vs. +0.23 at Day 14 and −1.11 vs. +0.30 at Day 28. For Sleep Disturbances, the FBCS group improved by −0.41 vs. −0.05 at Day 14 and −0.55 vs. +0.14 at Day 28. Lastly, Daytime Function scores decreased by −0.73 vs. −0.07 at Day 14 and −0.95 vs. −0.18 at Day 28.

**Conclusion:**

The FBCS demonstrated significant effectiveness in improving overall sleep outcomes among patients with primary insomnia. Moreover, the use of DHT enhanced patient adherence and facilitated reliable data collection.

**Clinical trial registration:**

https://www.chictr.org.cn/searchprojEN.html, identifier ChiCTR2100051187.

## Introduction

Insomnia is a prevalent clinical condition characterized by difficulty falling asleep or maintaining sleep, often accompanied by symptoms such as irritability or fatigue during waking hours. Among individuals receiving basic medical services, the incidence of insomnia can be as high as 50% (1), with approximately half being chronic cases (2), affecting various demographics including adolescents (3) and the older adult (4). Primary insomnia not only complicates treatment but also elevates the risk of depression and anxiety (5), cognitive dysfunction, and even suicide (6). *Cordyceps sinensis* (Berk.) Sacc. (called “Dongchongxiacao” in Chinese), as a precious natural herbal medicine, has been shown to have a positive impact on the treatment of respiratory (7, 8), liver (9), and kidney dysfunction (10), cardiopulmonary diseases (11) and inflammation (12), among others (13). It was approved by the National Medical Products Administration of China in 2021 as a food and drug homologous product (14), and has since gained increasing attention due to its nutritional and medicinal properties. *Cordyceps sinensis* contains a variety of bioactive components, including adenosine, cordycepin, mannitol, polysaccharide, and protein (15).

Cordycepin (3′-deoxyadenosine) is a natural pure compound extracted from *Cordyceps sinensis* (16) with a wide range of therapeutic potential, including antidepressant, analgesic, anti-inflammatory, anticancer, antiviral, and antifungal activities (17). It also aids in improving psychological disorders such as insomnia, irritability, and sleep disturbances (18, 19). The scientifically proven therapeutic effects of adenosine in treating sleep disorders and other central nervous system diseases stem from its role in sleep regulation (20), where it promotes sleep and maintains sleep homeostasis through endogenous neurotransmitter receptor mechanisms (21). Recent animal studies have shown that cordycepin increases nonrapid eye movement sleep via adenosine receptors in rats. Notably, after administration of cordycepin, the protein levels of adenosine receptors (A_1_, A_2A_, and A_2B_) were increased, especially in the rat hypothalamus, which plays an important role in sleep regulation (22).

Nevertheless, wild *Cordyceps sinensis* production is exceedingly rare, and its price has continued to rise in recent years (23). Overharvesting and ecological degradation have resulted in a significant decline in its natural yield (24). Additionally, ensuring the quality of bioactive ingredients in artificially cultivated *Cordyceps sinensis* remains challenging (25). *Cordyceps sinensis* produced through liquid fermentation technology, containing its active ingredient cordycepin, addresses the aforementioned issues (26). Recent studies have shown that functional beverages containing Cordyceps fermentation liquid have demonstrated reliable safety and health value (18, 27). In this study, the strains of the fermentation broth of *Cordyceps sinensis* (FBCS) were isolated from the fruit bodies of wild *Cordyceps sinensis* collected from the Zaqu Riverside in Yushu Prefecture, Qinghai Province, China.

In clinical research, there is a growing emphasis on non-invasive, non-interventional tools that help patients better understand their health conditions while addressing common research challenges. Studies indicate that one of the key barriers to successful clinical trial outcomes is patients’ insufficient understanding of clinical research and trial methodologies, as well as issues related to poor data quality (28–30). Digital Health Tools (DHT), such as our “Xingyun Family” application (App), play a vital role in addressing these challenges. “Xingyun Family” provides functionalities such as dietary and sleep tracking, online communication between patients and researchers, and structured data collection, enhancing patients’ adherence to research protocols. For researchers, “Xingyun Family” facilitates seamless data sharing through features like medication adherence tracking, symptom logging, and lifestyle monitoring, minimizing errors and improving trial efficiency.

**Figure 1:**
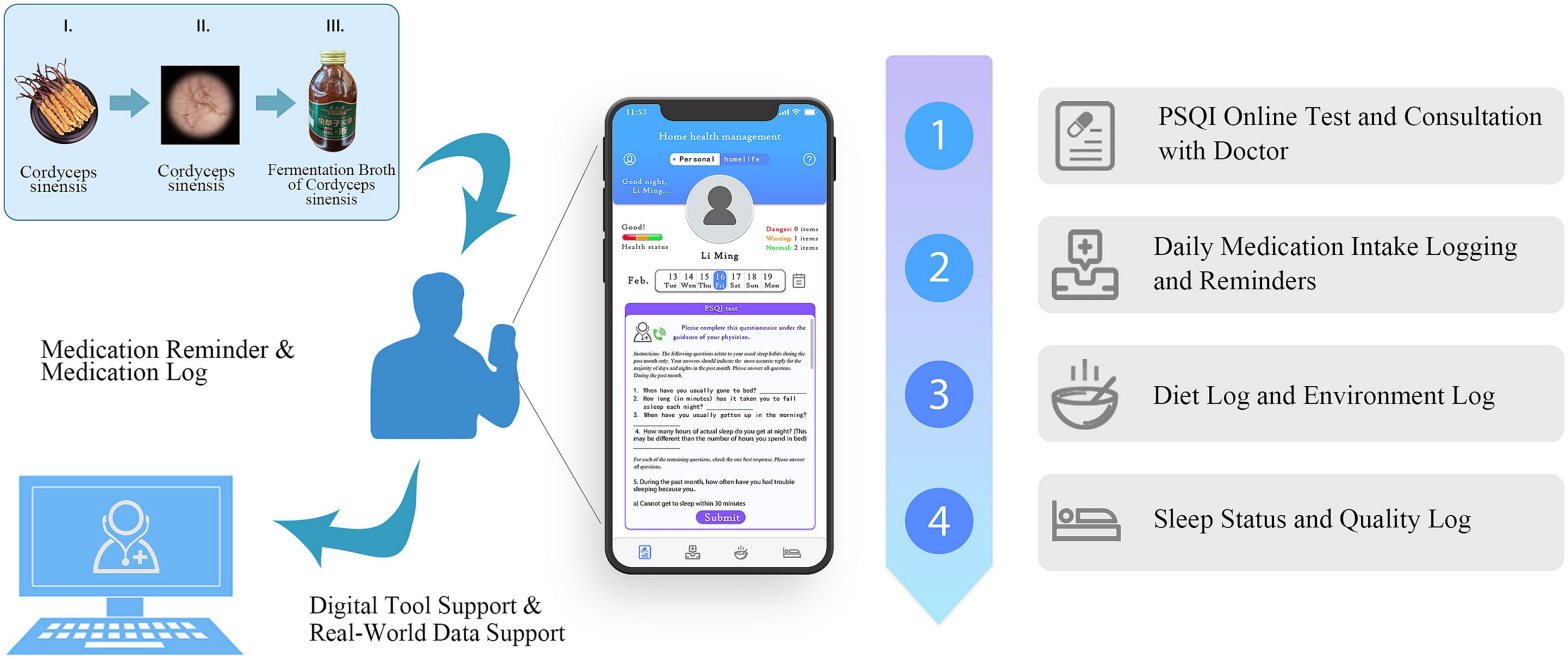
The overall structure diagram of this study.

Therefore, to prove that hypothesis and evaluate the effectiveness of FBCS in patients with primary insomnia, this study conducted a randomized controlled clinical trial, with the assistance of the DHT concepted smartphone App “Xingyun Family,” which was previously developed by our research group. Additionally, it explored the benefits of integrating DHT into clinical trials, such as improving patient compliance, enhancing data accuracy, and supporting the evaluation of traditional herbal treatments. The schematic structure of this study is depicted in Figure 1.

This trial was first registered in the Chinese Clinical Trial Registry with registration number ChiCTR2100051187, on 15/09/2021. This study was first authorized by the Affiliated Hospital of Chengdu University of Traditional Chinese Medicine on 09/07/2021, with the approval number 2021KL-011. Written informed consent was obtained from all participants prior to their enrollment.

## Materials and methods

### Study design

This study was conducted as a randomized, double-blind, placebo-controlled trial to assess the efficacy of the FBCS in patients with primary insomnia. The research assistant was responsible for enrolling participants and ensuring that all enrolled patients met the inclusion criteria. Ninety patients were enrolled and stratified by gender using a randomized method to ensure an equal distribution of men and women in both groups.

Basic patient information was recorded for the screening process, which includes name, gender, age, ID, weight, height, duration of insomnia, and medication history. Patients were instructed to refrain from consuming any other probiotic products or antibiotics and to abstain from caffeine or tea throughout the study period. To mitigate bias caused by anxiety, the Self-Rating Anxiety Scale (SAS) was utilized to exclude patients exhibiting symptoms of anxiety.

A Pittsburgh Sleep Quality Index (PSQI) score was recorded using the ‘Xingyun Family’ App at baseline, Day 14, and Day 28. The scale comprises 19 individual items that yield seven ‘component’ scores: Sleep Quality (SQua), Sleep Onset Latency (SOLat), Sleep Duration (SDur), Sleep Efficiency (SEff), Sleep Disturbances (SDis), Use of Sleep Medications (USMed), and Daytime Function (DFun). These components are utilized for self-assessment of sleep quality and disturbances within a one-month period (31), exhibiting high test–retest reliability and efficacy in primary insomnia (32). Additionally, it includes the evaluation of nocturnal sleep quality and daytime functioning, offering a comprehensive and effective means of assessing the degree of sleep improvement in clinical patients. The scale has been translated into multiple languages and its effectiveness validated (33–37).

**Figure 2:**
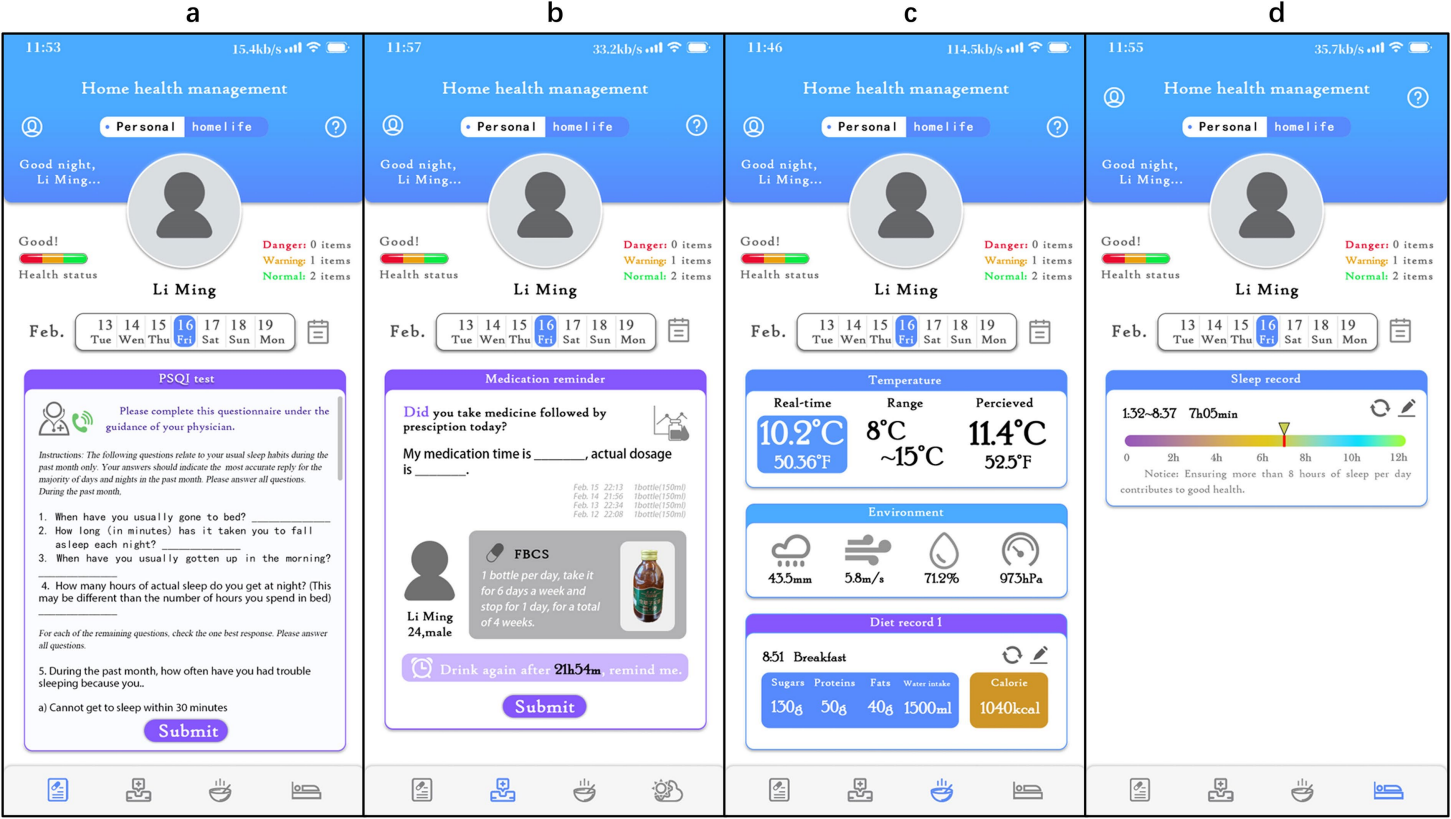
Detailed interface of the ‘Xingyun Family’ digital health tools app. **(A)** PSQI online test and consultation with a doctor; **(B)** daily medication intake logging and reminders; **(C)** diet log and environment log; **(D)** sleep status and quality log.

In the study, the “Xingyun Family” App was used to supervise the participants (Figure 2) and record diary entries every day after consuming the beverage, including information about whether, the time of consumption, adverse reactions, and taste experiences. In addition, participants were required to clock in daily to record their sleep time, living environment, and diet, as well as fill in the scale scores at specific points in the study. If necessary, participants could seek online consultations with doctors through the App. All the participants completed the trial at home.

### Participants

The participants, ranging in age from 20 to 75 years old and of any gender, were primarily diagnosed with primary insomnia, in accordance with the Chinese Classification and Diagnosis Criteria of Mental Disorders (3rd Edition): (1) Insomnia was nearly the sole symptom, with other symptoms being secondary to insomnia; (2) Insomnia occurred at least 3 times per week for more than 1 month; (3) Daytime dysfunction caused by insomnia, or as part of the symptoms of a mental disorder, interfered with social functioning; (4) Participants did not exhibit any physical or mental illnesses; (5) The PSQI score was > 5, while the SAS score was < 50.

Key exclusion criteria encompassed meeting the diagnosis of primary insomnia, to exclude (1) secondary insomnia caused by mental illness, abdominal pain, cancer pain, hiccups, and other physical diseases or organic brain lesions; (2) individuals dependent on alcohol, sedatives, hypnotics, and other medications (having not taken sleep-inducing drugs in the past month); (3) the presence of serious heart, liver, renal insufficiency, or other severe systemic diseases, as well as in pregnant or breastfeeding women; (4) participants with mental and neurological disorders unable to communicate normally; (5) meeting the criteria of PSQI ≤ 5 points or SAS ≥ 50 points.

The experimental sample in this age group encompasses young and middle-aged individuals with a high prevalence of insomnia (ages 20–60) as well as a portion of the older adult population (over 60 years). The former is influenced by work-related stress and circadian rhythm disturbances, while the latter is associated with the degeneration of age-related sleep regulatory mechanisms. To minimize the impact of confounding factors and ensure an adequate number of subjects across various age groups, we excluded adolescent patients under 20 years and older adult patients over 75 years. Furthermore, while females constituted 67.05% of the cohort, baseline gender distribution was balanced between groups (χ^2^ = 0.001, *p* = 1.000, refer to Table 1).

**Table 1:** Baseline demographic data (mean ± SE).

Variable	ALL (*n* = 88)	CONTROL (*n* = 44)	FBCS (*n* = 44)	*p*-value^a^
Age (year)	37.60 ± 1.66	38.25 ± 2.21	36.95 ± 2.48	0.366
Gender (female/male)	29/59	15/29	14/30	1.000
CD (month)	38.25 ± 5.66	42.07 ± 8.19	34.43 ± 7.85	0.919
EL (year)	15.07 ± 0.32	14.45 ± 0.51	15.68 ± 0.38	0.071
BMI	22.37 ± 0.37	22.53 ± 0.51	22.21 ± 0.53	0.542
SAS	40.01 ± 0.57	39.75 ± 0.82	40.27 ± 0.81	0.517
PSQI	11.19 ± 0.24	10.89 ± 0.30	11.50 ± 0.38	0.310
SQua	2.09 ± 0.06	2.02 ± 0.09	2.16 ± 0.08	0.268
SOLat	2.57 ± 0.07	2.52 ± 0.10	2.61 ± 0.10	0.337
SDur	1.98 ± 0.09	2.02 ± 0.13	1.93 ± 0.13	0.650
SEff	1.69 ± 0.13	1.66 ± 0.19	1.73 ± 0.18	0.826
SDis	1.38 ± 0.06	1.32 ± 0.07	1.43 ± 0.09	0.391
DFun	1.49 ± 0.09	1.34 ± 0.13	1.64 ± 0.14	0.115

^a^Group differences in gender were analyzed using the chi-square test, while other continuous variables were compared using the Mann–Whitney *U* test due to non-normal distributions. CD = Course of Disease; EL = Education Level; BMI = Body Mass Index; SAS = Self-Rating Anxiety Scale; PSQI = Pittsburgh Sleep Quality Index; SQua = Sleep Quality; SOLat = Sleep Onset Latency; SDur = Sleep Duration; SEff = Sleep Efficiency; SDis = Sleep Disturbances; DFun = Daytime Function.

The sample size determination was based on previous randomized controlled trials investigating the use of Cordyceps militaris for insomnia, as well as commonly accepted effect sizes observed in sleep intervention studies (38). Under the assumptions of a two-sided significance level of *α* = 0.05 and statistical power of 80%, the planned sample size was set at 45 participants per group (1: 1 allocation ratio), balancing methodological rigor with practical feasibility in the context of an exploratory clinical study.

A total of 90 patients with primary insomnia were recruited between September 16, 2021, and December 20, 2022. The participants underwent screening and diagnosis for insomnia by two clinically experienced physicians and were enrolled after providing informed consent.

### Interventions

The interventions in this study were FBCS or a placebo. The placebo was also a liquid beverage similar in appearance and taste but without the FBCS. Apart from the absence of the Cordyceps fermentation broth, the placebo contained the same excipients as the intervention beverage, including flavoring agents, sweeteners, and preservatives to match the taste, as well as the same color and packing bottle to match the appearance, ensuring the integrity of the blinding procedure. The FBCS and the placebo were manufactured and provided by Qinghai Mingyuewang Biotechnology Co., Ltd., with specifications of 150 ml per bottle.

The experimental group received treatment with FBCS, while the control group received treatment with a placebo. The dosage was one bottle per day (there was no requirement to take it at any specific time of day), taken for 6 days a week (7 days), with a one-day break. This treatment regimen was continued for Day 28, totaling 24 bottles. Both kinds of beverages were labeled with traceability codes and batch information, which remained confidential until database unlocking.

The experiment selected a fixed dosage of 150 ml/day based on the following considerations: Firstly, preliminary animal experiments conducted by Hu et al. (22), based on the content of the product, the intake of cordycepin at this dosage (<16.5 mg/kg) is significantly below the safety threshold (LD50 > 2000 mg/kg), with no reported abnormalities in liver or kidney function. A fixed single dosage effectively mitigates the risk of adverse events arising from participants inadvertently self-administering excessive doses due to unclear communication. Secondly, administering one bottle orally once a day simplifies the medication regimen. Coupled with the medication reminder function of the “Xingyun Family” APP, patient compliance rates in the study reached over 98%. Thirdly, as an exploratory randomized controlled trial (RCT), employing a single dosage minimizes biases resulting from variations in dosage, frequency of administration, or individual habits.

Participants were randomly assigned into two groups using stratified randomization to ensure balanced distribution of factors such as gender and age, thereby improving the trial’s validity and reliability. An independent data manager generated the random allocation sequence using the App, with only the data manager aware of the results. Identical labels and packaging were used for FBCS and placebo. Participants, researchers, and authorized personnel from the sponsor were blinded to the treatment allocation. The interventions were assigned by personnel unaware of the randomization sequence and allocation process, ensuring the trial’s double-blind nature.

Both sets of beverages were packaged identically and tasted similar, making them indistinguishable. However, researchers could access group contents in case of a medical emergency. No breaches of blinding occurred during the study. Throughout the trial, participants, healthcare providers, and trial staff remained blinded to treatment allocation.

### Efficacy endpoints

The efficacy endpoint of this study was the mean change in the total score of the PSQI and its subscales (SQua, SOLat, SDur, SEff, SDis, DFun, USMed) from the baseline period before treatment to the 14th and Day 28 s of treatment, with lower scores indicating fewer insomnia symptoms. During the baseline period and after the 14th and Day 28 s of treatment, researchers needed to promptly assess the sleep changes of the participants through the App and conduct a comprehensive evaluation of daytime function and nocturnal sleep using the PSQI scale.

### Data collection

Participants were instructed to complete a daily sleep diary using a mobile application, recording their actual sleep and wake times. These time points were used solely for monitoring participant compliance and were not included in efficacy assessments. At the predefined efficacy evaluation time points (Day 14 and Day 28), study staff contacted participants by telephone to guide them in completing the PSQI assessment within the application. During this process, the study staff simultaneously recorded a parallel copy of the PSQI scores for verification purposes. Both sets of scoring data were cross-checked during the final data collation and statistical analysis stages to ensure data accuracy and to minimize potential bias arising from self-reported outcomes.

### Data statistics and study parameters

The primary statistical approach used in this study was a two-way mixed-design ANOVA to assess the main effects of time, group, and their interaction on PSQI total and component scores across three time points (Day 0, Day 14, and Day 28). This analysis was also applied in subgroup analyses stratified by sex and age to explore differential intervention effects. To complement the ANOVA results and verify specific changes at different time points, additional non-parametric tests were conducted. Between-group comparisons were performed using the Mann–Whitney *U* rank sum test, and within-group comparisons were conducted using the Wilcoxon paired rank sum test when normality or homogeneity of variance assumptions were not met. Independent samples *t*-tests and paired t-tests were applied when assumptions were satisfied. Quantitative data were summarized as mean ± standard error (SE). Qualitative data were described using frequency and percentage (%) and compared using the Chi-square test (χ^2^). Bonferroni correction was applied to primary outcomes (PSQI total score and 6 subscales), adjusting the significance threshold to *α* = 0.007 (0.05/7 comparisons). A significance level of α = 0.007 was adopted (not used in baseline data), and *P* < α was considered statistically significant. All data were managed using Microsoft Excel 2022 (Microsoft Corporation, Redmond, WA, USA) and analyzed with IBM SPSS Statistics for Windows, Version 26.0 (IBM Corp., Armonk, NY, USA).

## Results

### Participant flow

**Figure 3:**
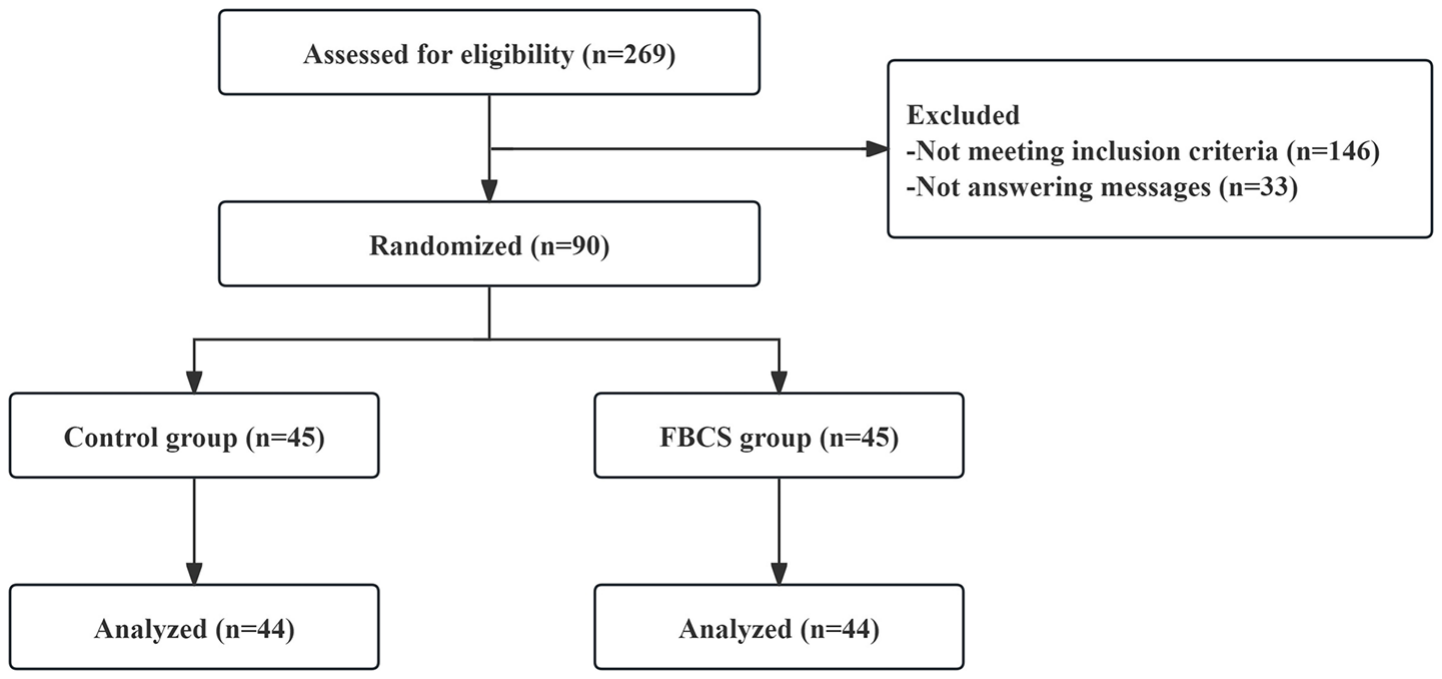
The flow diagram of study selection.

Registration commenced on September 16, 2021, and concluded on December 20, 2022 (of which, the first patient was enrolled on October 26, 2021). Among the 269 consulted, 146 did not meet the inclusion criteria, while 33 ceased responding, resulting in 90 patients passing the screening criteria. At the conclusion of the 28-day trial period, one participant from the FBCS group discontinued (due to taking sleeping drugs), and another from the control group withdrew consent. Subsequently, 88 successfully completed cases (44 in the FBCS group and 44 in the control group) were included in the final analysis. The blinding procedure was confirmed through drink tracing images received by the participants and their guesses. The compliance rate of the participants exceeded 98%. No harmful events associated with the beverage were reported. The participant flow chart is depicted in Figure 3.

### Baseline data

**Figure 4:**
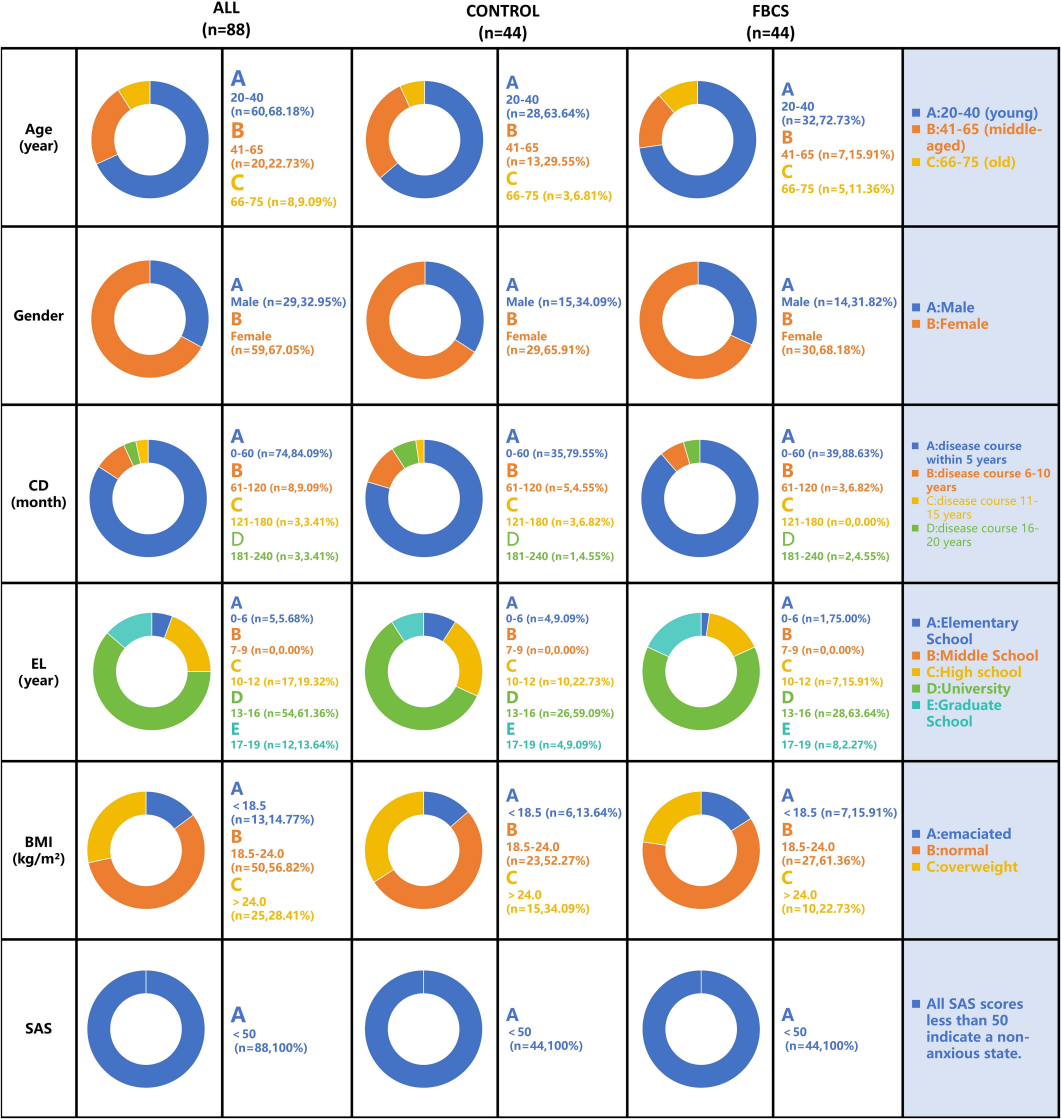
Baseline demographic characteristics distribution chart. The results are depicted in the figure below, expressed as mean ± SE. Gender is represented by participant numbers. CD = Course of Disease; EL = Education Level; BMI = Body Mass Index; SAS = Self-Rating Anxiety Scale.

The baseline characteristics of the PSQI scale for both groups are summarized in Table 1. The demographic characteristics and baseline features, along with the proportions of the two groups, are presented in Figure 4. Demographic and baseline characteristics were well-balanced between the two groups (*p* > 0.05 for all comparisons; Table 1 and Figure 4). Among the 88 participants in the study, 59 were female and 29 were male, with ages ranging from 20 to 75 years. All participants were diagnosed with primary insomnia, with a mean Insomnia Severity Index score of 40.01 ± 0.57 at baseline. The PSQI score was 11.19 ± 0.24 at baseline, primarily reflecting difficulties in falling asleep and early morning awakenings. None of the participants were taking sleep medication at baseline.

### Outcomes of the FBCS intervention on overall sleep

In this study, the effectiveness of FBCS and placebo in improving overall sleep status among patients with primary insomnia was evaluated using a two-way mixed-design ANOVA. This analysis assessed the main effects of time and group, as well as their interaction, on the total PSQI score and its seven component scores across three time points: Day 0, Day 14, and Day 28.

**Figure 5:**
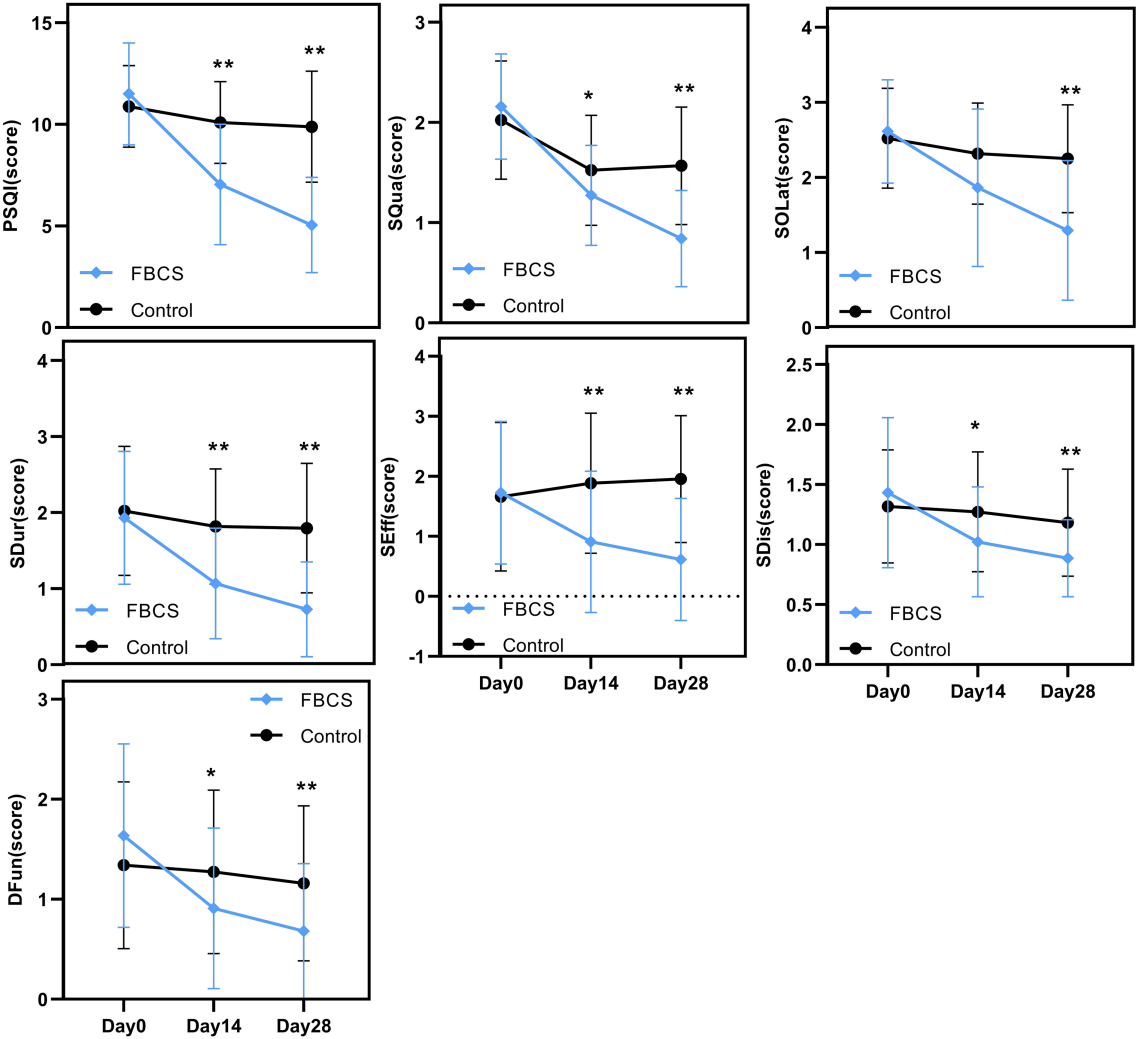
Between-group trends over time. Between-group comparisons were conducted using the Mann–Whitney U test. * *p* < 0.05, ** *p* < 0.01. PSQI = Pittsburgh Sleep Quality Index; SQua = Sleep Quality; SOLat = Sleep Onset Latency; SDur = Sleep Duration; SEff = Sleep Efficiency; SDis = Sleep Disturbances; DFun = Daytime Function.

**Figure 6:**
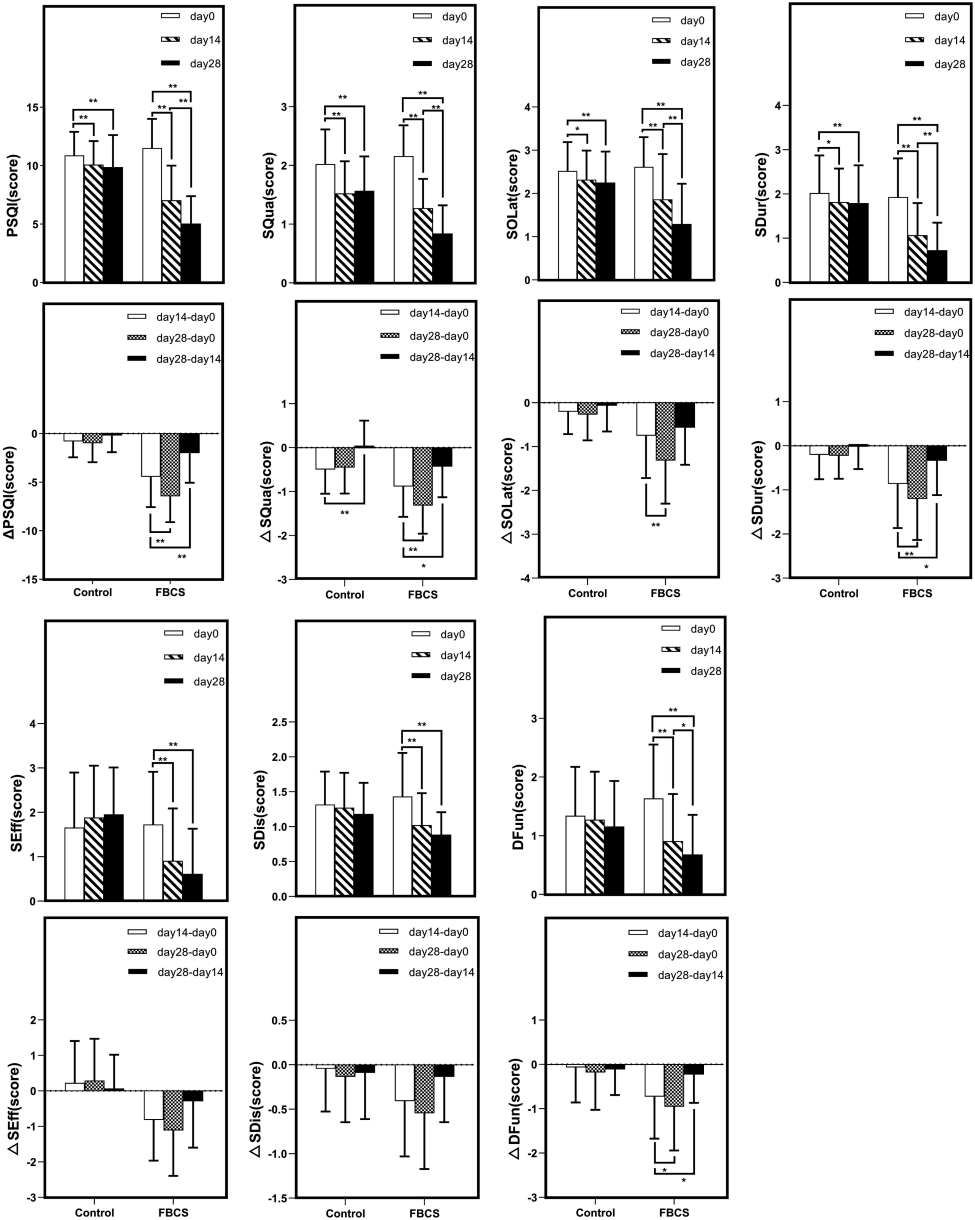
Within-group trends over time. Within-group comparisons were conducted using the Wilcoxon signed-rank test. * *p* < 0.05, ** *p* < 0.01. *Δ* indicates the change scores calculated between different time points: Δ = Day 14 - Day 0, Day 28 - Day 14, or Day 28 – Day 0. PSQI = Pittsburgh Sleep Quality Index; SQua = Sleep Quality; SOLat = Sleep Onset Latency; SDur = Sleep Duration; SEff = Sleep Efficiency; SDis = Sleep Disturbances; DFun = Daytime Function.

The results of the mixed-design ANOVA are summarized in Table 2. Estimated marginal means of PSQI scores at each time point, along with within-group changes and between-group differences, are presented in Table 3. The temporal trends of sleep improvement and the group-by-time interactions are visually illustrated in Figures 5, 6.

**Table 2:** Results of two-way mixed-design ANOVA on PSQI total and component scores.

Outcomes	Effect	*F*	*df*	*p*-value	*η^2^*
PSQI	Time	109.087	2.000	<0.001	0.559
	Group	31.994	1.000	<0.001	0.271
	Time × Group	57.480	2.000	<0.001	0.401
SQua	Time	98.066	2.000	<0.001	0.533
	Group	10.689	1.000	0.002	0.111
	Time × Group	21.121	2.000	<0.001	0.197
SOLat	Time	47.489	2.000	<0.001	0.356
	Group	9.575	1.000	0.003	0.100
	Time × Group	20.250	2.000	<0.001	0.191
SDur	Time	44.252	2.000	<0.001	0.340
	Group	20.590	1.000	<0.001	0.193
	Time × Group	19.849	2.000	<0.001	0.188
SEff	Time	5.659	2.000	0.004	0.062
	Group	14.662	1.000	<0.001	0.146
	Time × Group	16.977	2.000	<0.001	0.165
SDis	Time	17.664	2.000	<0.001	0.170
	Group	3.542	1.000	0.063	0.040
	Time × Group	7.369	2.000	0.001	0.079
DFun	Time	22.689	1.677	<0.001	0.209
	Group	1.688	1.000	0.197	0.019
	Time × Group	11.614	1.677	<0.001	0.119

PSQI = Pittsburgh Sleep Quality Index; SQua = Sleep Quality; SOLat = Sleep Onset Latency; SDur = Sleep Duration; SEff = Sleep Efficiency; SDis = Sleep Disturbances; DFun = Daytime Function.

**Table 3:** Within-group and between-group comparison *p*-values^a^.

Outcomes	Within-Group comparison	CONTROL		FBCS		Between-Group comparison	*p*-value^d^	Mean change^f^
		*p*-value^b^	Mean change^e^	*p*-value^c^	Mean change^e^			
PSQI	Day 0/Day 14	0.003	−0.80	<0.001	−4.45	Day 0	0.365	+0.61
	Day 14/Day 28	0.417	−0.20	<0.001	−2.00	Day 14	<0.001	−3.05
	Day 0/Day 28	0.001	−1.00	<0.001	−6.45	Day 28	<0.001	−4.84
SQua	Day 0/Day 14	<0.001	−0.50	<0.001	−0.89	Day 0	0.268	+0.14
	Day 14/Day 28	0.593	+0.05	0.001	−0.43	Day 14	0.019	−0.25
	Day 0/Day 28	<0.001	−0.45	<0.001	−1.32	Day 28	<0.001	−0.73
SOLat	Day 0/Day 14	0.013	−0.20	<0.001	−0.75	Day 0	0.337	+0.09
	Day 14/Day 28	0.439	−0.07	<0.001	−0.57	Day 14	0.053	−0.45
	Day 0/Day 28	0.005	−0.27	<0.001	−1.32	Day 28	<0.001	−0.95
SDur	Day 0/Day 14	0.020	−0.20	<0.001	−0.86	Day 0	0.650	−0.09
	Day 14/Day 28	0.763	−0.02	0.007	−0.34	Day 14	<0.001	−0.75
	Day 0/Day 28	0.008	−0.23	<0.001	−1.20	Day 28	<0.001	−1.07
SEff	Day 0/Day 14	0.195	+0.23	<0.001	−0.82	Day 0	0.826	+0.07
	Day 14/Day 28	0.597	+0.07	0.121	−0.30	Day 14	<0.001	−0.98
	Day 0/Day 28	0.090	+0.30	<0.001	−1.11	Day 28	<0.001	−1.34
SDis	Day 0/Day 14	0.527	−0.05	<0.001	−0.41	Day 0	0.391	+0.11
	Day 14/Day 28	0.248	−0.09	0.083	−0.14	Day 14	0.017	−0.25
	Day 0/Day 28	0.083	−0.14	<0.001	−0.55	Day 28	0.001	−0.30
DFun	Day 0/Day 14	0.623	−0.07	<0.001	−0.73	Day 0	0.115	+0.30
	Day 14/Day 28	0.197	−0.11	0.025	−0.23	Day 14	0.048	−0.36
	Day 0/Day 28	0.102	−0.18	<0.001	−0.95	Day 28	0.004	−0.48

^a^The *p*-value results are reported to three decimal places. ^b^*P*-Value of Within-Group Comparison for the Control Group. ^c^*p*-Value of Within-Group Comparison for the FBCS Group. ^d^*P*-Value of Between-Group Comparison for the Control and FBCS Groups. ^e^Within-group: Mean Change = Day14– Day 0, Day28–Day14, Day28– Day 0. ^f^Between-group: FBCS − CONTROL at Day 0, Day 14, and Day 28. Abbreviations: PSQI = Pittsburgh Sleep Quality Index; SQua = Sleep Quality; SOLat = Sleep Onset Latency; SDur = Sleep Duration; SEff = Sleep Efficiency; SDis = Sleep Disturbances; DFun = Daytime Function.

A two-way mixed-design ANOVA revealed significant main effects of time, group, and time × group interaction on PSQI, SQua, SOLat, SDur, and SEff (all *p* < 0.01). As shown in Figure 5, scores for these outcomes declined steadily from Day 0 to Day 28, especially in the FBCS group, indicating improved sleep quality, sleep onset latency, sleep duration, and sleep efficiency over time. For SDis and DFun, the main effect of time (*p* < 0.01) and time × group interaction (*p* < 0.01) were also significant, while the group effect was not significant (SDis: *p* = 0.063; DFun: *p* = 0.197). Both outcomes showed downward trends, suggesting time-related improvement, with greater changes observed in the FBCS group.

Between-group comparisons at Day 14 showed that, except for SOLat (*p* = 0.053), the FBCS group had significantly greater average reductions than the control group in PSQI total score (mean change: −4.45 vs. –0.80; between-group difference = 3.05, *p* < 0.01), SQua (−0.89 vs. –0.50; difference = 0.25, *p* < 0.05), SDur (−0.86 vs. –0.20; difference = 0.75, *p* < 0.01), SEff (−0.82 vs. +0.23; difference = 0.98, *p* < 0.01), SDis (−0.41 vs. –0.05; difference = 0.25, *p* < 0.05), and DFun (−0.73 vs. –0.07; difference = 0.36, *p* < 0.05). Although the between-group difference in SOLat did not reach significance, the FBCS group still showed a greater average improvement (−0.75 vs. –0.20).

At Day 28, the FBCS group demonstrated significantly greater mean changes across all PSQI components compared to the control group. The mean change in the total PSQI score was −6.45 in the FBCS group versus −1.00 in the control group (between-group difference = 4.84, *p* < 0.01). For each component, the FBCS group showed larger mean reductions than the control group: SQua (−1.32 vs. –0.45; difference = 0.73, *p* < 0.01), SOLat (−1.32 vs. –0.27; difference = 0.95, *p* < 0.01), SDur (−1.20 vs. –0.23; difference = 1.07, *p* < 0.01), SEff (−1.11 vs. +0.30; difference = 1.34, *p* < 0.01), SDis (−0.55 vs. +0.14; difference = 0.30, *p* < 0.05), and DFun (−0.95 vs. –0.18; difference = 0.48, *p* < 0.01). Comparing Day 28 to Day 14, the FBCS group showed a significant further reduction in the total PSQI score (*p* < 0.01), while no significant change was observed in the control group. Between-group differences in SDur, SEff, SDis, and DFun from Day 14 to Day 28 were not statistically significant. However, the FBCS group continued to show significant improvements in SQua and SOLat (*p* < 0.01), whereas the control group did not.

Within-group comparisons showed that both groups experienced significant improvements in PSQI total score and SQua at Day 14 relative to baseline (*p* < 0.01). However, only the FBCS group exhibited significant average reductions in SOLat and SDur (*p* < 0.01); no significant changes were seen in these components in the control group. Additionally, significant within-group reductions were observed in SEff, DFun, and SDis in the FBCS group (*p* < 0.01), but not in the control group.

At Day 28, both groups again showed significant reductions in PSQI total score, SQua, SOLat, and SDur compared to baseline (*p* < 0.01). The FBCS group also demonstrated significant within-group reductions in SEff, DFun, and SDis (*p* < 0.01), while these three measures remained unchanged in the control group.

The PSQI score included a comparison of sleep medication use. However, to avoid the potential effects of sedative hypnotics on the study results, participants were required to abstain from taking any sleeping pills for the month preceding the study. Therefore, no sleep medication was used by participants in either group in this study.

Based on the above comparisons, it can be concluded that the FBCS intervention led to significantly greater and more consistent improvements in overall sleep status compared to placebo. These effects were observed as early as Day 14 and further strengthened by Day 28, supporting the efficacy of FBCS in improving multiple dimensions of sleep among patients with primary insomnia.

### Gender-stratified outcomes of the FBCS intervention on overall sleep

As shown in Table 4, a mixed-design ANOVA was conducted separately for male and female participants. In both subgroups, significant main effects of time were found for PSQI, SQua, SOLat, and SDur (all *p* < 0.01), with significant time × group interactions also observed (all *p* < 0.01), indicating greater improvements in the FBCS group over time. However, the group effect for SOLat in males was not significant (*p* = 0.296), suggesting no between-group difference at the overall level in this component.

**Table 4:** Results of two-way mixed-design ANOVA on PSQI total and component scores by sex.

Outcomes	Effect	*F*		*df*		*p*-value		η^2^	
		Male	Female	Male	Female	Male	Female	Male	Female
PSQI	Time	42.437	66.161	2.000	2.000	<0.001	<0.001	0.611	0.537
	Group	8.853	23.346	1.000	1.000	0.006	<0.001	0.247	0.291
	Time × Group	17.104	40.124	2.000	2.000	<0.001	<0.001	0.388	0.413
SQua	Time	31.745	66.849	2.000	2.000	<0.001	<0.001	0.540	0.540
	Group	7.081	5.577	1.000	1.000	<0.001	0.022	0.208	0.089
	Time × Group	6.928	13.922	2.000	2.000	0.002	<0.001	0.204	0.196
SOLat	Time	20.136	29.288	2.000	2.000	<0.001	<0.001	0.427	0.339
	Group	1.137	11.800	1.000	1.000	0.296	0.002	0.040	0.159
	Time × Group	12.980	10.074	2.000	2.000	<0.001	<0.001	0.325	0.150
SDur	Time	34.491	19.775	2.000	2.000	<0.001	<0.001	0.561	0.258
	Group	4.638	15.778	1.000	1.000	0.040	<0.001	0.147	0.217
	Time × Group	15.893	9.008	2.000	2.000	<0.001	<0.001	0.371	0.136
SEff	Time	1.680	4.323	2.000	2.000	0.196	0.016	0.059	0.070
	Group	1.884	13.030	1.000	1.000	0.181	0.001	0.065	0.186
	Time × Group	2.370	17.806	2.000	2.000	0.103	<0.001	0.059	0.238
SDis	Time	2.821	15.540	2.000	2.000	0.068	<0.001	0.095	0.214
	Group	1.132	2.338	1.000	1.000	0.297	0.132	0.040	0.039
	Time × Group	0.721	7.376	2.000	2.000	0.491	0.001	0.026	0.115
DFun	Time	24.117	7.690	2.000	1.749	<0.001	0.001	0.472	0.119
	Group	0.051	1.838	1.000	1.000	0.823	0.181	0.002	0.031
	Time × Group	3.377	8.801	1.553	1.749	0.055	0.001	0.111	0.134

PSQI = Pittsburgh Sleep Quality Index; SQua = Sleep Quality; SOLat = Sleep Onset Latency; SDur = Sleep Duration; SEff = Sleep Efficiency; SDis = Sleep Disturbances; DFun = Daytime Function.

Sex-related differences were observed in SEff, SDis, and DFun. Among males, only DFun showed a significant time effect (*p* < 0.01), while no other significant effects were found (all *p* > 0.05), indicating limited responsiveness. In contrast, females showed significant effects in SEff and SDis, with most effects reaching *p* < 0.01, except for the group effect in SDis (*p* = 0.04). For DFun, significant time and interaction effects were observed (both *p* < 0.01), while the group effect was not significant (*p* = 0.181).

These results suggest that female participants may have experienced broader and more consistent improvements in sleep-related outcomes compared to males during the intervention period.

### Age-stratified outcomes of the FBCS intervention on overall sleep

For young adults aged 20–40 years, significant effects (*p* < 0.05) were found in PSQI, SQua, SOLat, SDur, SEff, SDis, and DFun. Non-significant results were observed in the time effect of SEff (*p* = 0.087), the group effect of SDis (*p* = 0.272) and the group effect of DFun (*p* = 0.107).

For middle-aged adults aged 41–65 years, significant effects (*p* < 0.05) were found in PSQI, SQua, SOLat, SDur, SEff, and SDis. Non-significant results were observed in the group effects of PSQI (*p* = 0.117), SQua (*p* = 0.602), and SOLat (*p* = 0.608); the group and interaction effects of SDur (*p* = 0.122, 0.096); the group effect of SEff (*p* = 0.253); the group effect of SDis (*p* = 0.076); and all effects of DFun (*p* = 0.177, 0.460, 0.103).

For older adults aged 66–75 years, significant effects (*p* < 0.05) were found in PSQI, SQua, SOLat, SDur, SEff, and DFun. Non-significant results were observed in the interaction effect of PSQI (*p* = 0.058); the group effect of SQua (*p* = 0.139); the group and interaction effects of SOLat (*p* = 0.068, 0.442); the time and interaction effects of SDur (*p* = 0.080); the time and interaction effects of SEff (*p* = 0.375); all effects of SDis (*p* = 0.060, 0.818, 0.756); and the group effect of DFun (*p* = 0.550).

**Figure 7:**
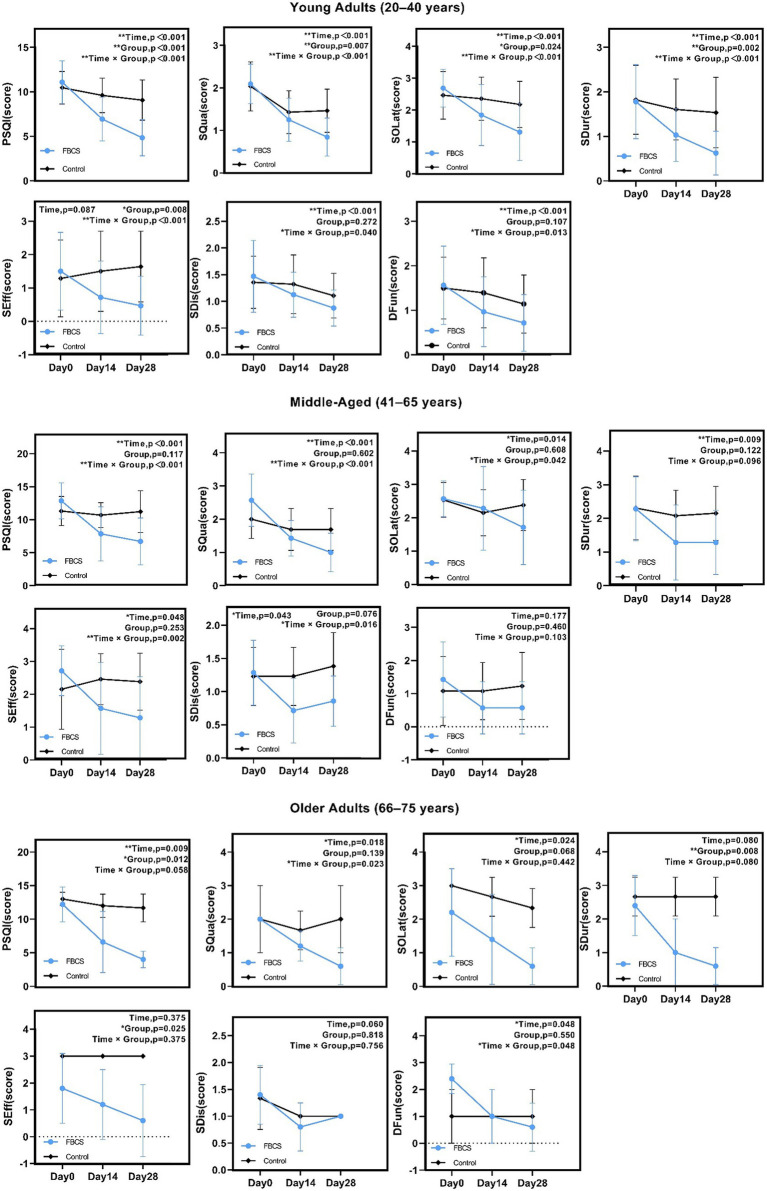
Results of two-way mixed-design ANOVA on PSQI total and component scores by age group. *p < 0.05, **p < 0.01. PSQI = Pittsburgh Sleep Quality Index; SQua = Sleep Quality; SOLat = Sleep Onset Latency; SDur = Sleep Duration; SEff = Sleep Efficiency; SDis = Sleep Disturbances; DFun = Daytime Function.

Overall, sleep improvements were observed across all age groups, with young adults showing the strongest and most consistent effects, followed by moderate effects in middle-aged adults and relatively limited effects in older adults. These results suggest a declining responsiveness to the intervention with increasing age. Detailed age-stratified results are presented in Figure 7.

### Adverse events

We have implemented an adverse reaction reporting feature in the “Xingyun Family” application, allowing patients to document adverse reaction information and establish immediate contact with their supervising physician. Among the 44 participants in the FBCS group, three minor adverse events were reported, all involving more frequent defecation (less than three times per day, excluding watery stool), which returned to normal within 1–3 days. It is unclear whether these reactions were caused by the tested beverage or other factors.

The active component Cordycepin in *Cordyceps sinensis* has demonstrated beneficial regulatory effects on the gastrointestinal system. Studies have shown that it can alleviate constipation through multiple mechanisms, including enhancing the integrity of the intestinal mucosal barrier, reducing metabolism-associated inflammation, and modulating gut microbiota composition (39, 40). Therefore, the transient increase in bowel movements observed in this study may represent a physiological response during the gut-regulating process of Cordycepin, suggesting its potential positive role in gastrointestinal functional modulation.

Adverse events observed in this study primarily occurred within the first week of administration and did not persist throughout the treatment period. This pattern of early-phase, mild gastrointestinal symptoms suggests a possible adaptation response or individual sensitivity rather than a clear time-dependent or cumulative dose-related effect. Although a fixed-dose protocol was employed and no pharmacokinetic or multi-dose analyses were conducted, the potential for dose-dependent mechanisms cannot be entirely ruled out. Nonetheless, the overall safety profile of Cordycepin appears favorable in this trial. Future studies may benefit from incorporating dose-ranging strategies and dynamic biomarker assessments to further clarify the safety margins and mechanistic basis of any adverse responses.

## Discussion

This study primarily focused on patients with non-organic and non-psychiatric primary insomnia, thereby excluding the confounding factors of secondary insomnia caused by other diseases. This study preliminarily investigated the effects of FBCS on primary insomnia. The results showed that participants in the FBCS group experienced fewer sleep disturbances, reduced time to fall asleep, improved sleep quality, increased sleep duration, higher sleep efficiency, and reduced daytime dysfunction.

### Improvement of primary insomnia through FBCS

Previous studies have shown that sleep disorders are closely related to fatigue (41). *Cordyceps sinensis* has been found to enhance the body’s anti-fatigue capabilities (42), and resist stress and improve work performance (43). These findings are consistent with the results of our study, where FBCS produced significant improvements in overall sleep quality, particularly by Day 28. Notably, we observed marked improvements as early as Day 14, which were further enhanced by Day 28, suggesting both an early onset and a sustained or cumulative effect. The initial response by Day 14 may reflect the short-term regulatory impact of FBCS on sleep–wake physiology and fatigue modulation, while the continued improvement by Day 28 indicates the presence of cumulative effects during continuous administration and a sustained improvement in sleep quality.

In sleep research, adenosine is an endogenous substance that promotes sleep by binding to adenosine A_1_ and A_2A_ receptors (21). Studies have shown that the administration of adenosine analogs or adenosine metabolism inhibitors can play a role in sleep homeostasis (44). Cordycepin, an adenosine analog, has the potential to improve sleep by acting on these adenosine receptors, particularly in enhancing sleep quality and restoring normal sleep patterns. This mechanism aligns with our findings. The observed improvements were further supported by a two-way mixed-design ANOVA, which revealed significant main effects of time, group, and their interaction on total PSQI score and several sleep-related components, including sleep quality, latency, duration, and efficiency. These results highlight that the FBCS intervention not only exerted broad effects across sleep dimensions but also outperformed the placebo over time. Although SDis and DFun showed no significant group-level effects, the significant time × group interactions observed for these components suggest that the FBCS group experienced greater time-related improvements. The consistency between statistical trends and the temporal trajectory of estimated marginal means further reinforces the robustness of our findings.

*Cordyceps sinensis*, recognized as both a food and medicinal product, has been used for centuries in China and other Asian countries (45). It is known for its tonic and calming properties (15), making it a valuable traditional medicine. Recent studies have shown that cordycepin plays a significant role in improving cognitive function and providing neuroprotection (46). It achieves this by modulating adenosine A_1_ and A_2A_ receptors, with a notable reduction in the levels of adenosine A_2A_ receptors in the hippocampus (47). Theoretically, it can be inferred that cordycepin promotes sleep and inhibits excessive activation of the arousal system by modulating adenosine A_1_ and A_2A_ receptors, particularly through a significant reduction in adenosine A_2A_ receptor levels in the hippocampus. This inference is consistent with previous research, which suggests that adenosine A_1_ receptors promote sleep and sleep homeostasis, while adenosine A_2A_ receptors facilitate sleep by inhibiting the arousal system, thereby forming a unified sleep–wake regulation model (48). It also aligns with our experimental results, where FBCS is shown to improve sleep. The progressive improvements observed between Day 14 and Day 28 in our study support the notion that FBCS not only initiates but also consolidates improvements in sleep regulation over time.

A clinical randomized controlled trial has shown that *Cordyceps sinensis* combined with duloxetine yields significantly greater improvements in sleep quality among patients with depression compared to duloxetine combined with placebo (49), further supporting the potential clinical value of FBCS in insomnia intervention.

In the control group, significant improvements were observed in the PSQI total score and SQua on Day 14 compared to the baseline period. However, there were no significant differences in the scores of these two items on Day 28 compared to Day 14. This phenomenon may indicate that the temporary improvement in sleep status between baseline and Day 14 in the control group can be partially attributed to the placebo effect, reflecting the influence of patients’ trust in the study and their expectations on their perception (50). However, over time to Day 28, there were no significant differences in the all items in the control group compared to Day 14. The effects of the placebo were significantly weakened over time compared to the effects of the FBCS. Some studies have shown that the placebo effect largely depends on patients’ expectations of the treatment effect, with higher expectations leading to a greater placebo effect (51). Over time, patients’ expectations of the placebo may change, especially when their condition does not improve significantly, resulting in a decrease in the placebo effect. This is consistent with the results observed in the control group in this study. Meanwhile, on the Day 14, compared to the control group, the FBCS group not only had significant improvements in the PSQI total score and SQua, but also in SOLat, SDur, SEff, SDis, and DFun. Over time, by the Day 28, the FBCS group showed significant improvements compared to the control group in the PSQI total score, SQua, SOLat, SDur, SEff, SDis, and DFun.

Therefore, at the mid-stage of the study, by including a specific numerical assessment of the patients’ sleep scores on Day 14 of the mid-stage of the study and comparing them with the scores on Day 28 of the study, the potential influence of placebo effects on the experimental results can be identified and excluded.

To more sensitively capture individual-level changes induced by the intervention, this study adopted *Δ* values as primary effect indicators, in line with methodologies used in previous randomized controlled trials (52). Δ values were calculated based on the differences in scores between Day 0, Day 14, and Day 28, allowing quantification of changes across various time intervals. Compared to raw scores, Δ values provide a more direct representation of individual responsiveness and effectively control for baseline differences that may confound between-group comparisons. In this study, Δ values served as key statistical indicators to evaluate the multidimensional effects of FBCS on subjective sleep quality, sleep onset latency, sleep efficiency, and daytime functioning. The results showed that the FBCS group exhibited significantly greater improvements across multiple Δ metrics compared to the control group, thereby strengthening statistical support for the intervention effect and providing more clinically interpretable evidence of efficacy.

Subgroup analysis showed that both sexes benefited from the FBCS intervention, especially in PSQI, SQua, SOLat, and SDur. However, females exhibited broader improvements, with significant effects also observed in SEff, SDis, and DFun. This may reflect sex-specific differences in sleep regulation and neuroendocrine sensitivity—particularly given evidence linking estrogen to adenosine receptor-mediated sleep regulation (53). Such biological mechanisms may underlie the greater responsiveness seen in female participants, which aligns with the enhanced sensitivity observed in this study. Age-related differences were also apparent. Young adults showed consistent improvements across most components, while middle-aged adults exhibited moderate effects, and older adults responded less markedly. These differences may be related to age-associated changes in sleep homeostasis, receptor function, or neuroplasticity, potentially reducing FBCS effectiveness with age. Overall, these results suggest that individual factors such as sex and age influence responsiveness to FBCS and should be considered in future applications.

Such analytical methods allow us to draw a clear conclusion that consuming the FBCS positively affects patients’ sleep. In addition, the duration of this study was Day 28, which is comparable to the period used in clinical sleep quality evaluation trials (51, 52). Considering that primary insomnia typically lasts longer than 1 month, a 28-day treatment period is relatively reasonable.

### Role of DHT in enhancing patient engagement and data integrity in clinical trials

In clinical trials, patient compliance and data integrity are critical to the reliability of study outcomes. Integrating DHT into research protocols offers a non-invasive solution to overcoming challenges such as non-compliance, data inaccuracies, and inefficiencies in data collection. In this study, the “Xingyun Family” App supported health monitoring, adherence tracking, and structured data collection without intervening in treatment. A compliance rate exceeding 98% was achieved among the 90 enrolled patients, with only one participant withdrawing due to the use of sleeping pills. This is significantly higher than the adherence rates typically seen in traditional trials, which range from 50 to 80% (54–56), with dropout rates largely due to lack of reminders, protocol misunderstandings, and logistical challenges (57). Trials using manual follow-ups and paper-based data collection systems exhibit notably lower adherence compared to those employing digital tools for reminders and monitoring (58).

Additionally, digital data collection has been shown to reduce costs by 55%, with savings of 49–62% compared to paper-based methods (59). In this study, the App incorporated an electronic version of the PSQI, automating data collection, ensuring real-time updates, and minimizing errors. This process streamlined data management while providing timely medication reminders and personalized feedback, further enhancing adherence and understanding of trial protocols.

Unlike remote telemedicine, which involves direct therapeutic interventions, DHT such as the “Xingyun Family” App focus on health monitoring and data management (60). While telemedicine typically includes diagnosis and treatment by healthcare professionals, DHT enhance adherence and streamline data collection without directly affecting clinical outcomes (61, 62). By facilitating structured health monitoring and communication between patients and researchers, the App ensures that the therapeutic effects observed are attributed solely to the primary intervention—the Fermentation Broth of *Cordyceps sinensis* (FBCS). Through features like medication reminders, sleep tracking, dietary logging, adverse reaction recording, and online communication, the App demonstrated how DHT can improve patient engagement, adherence, and data integrity. These findings align with research by Seoul National University (63), which also emphasizes the role of mobile applications in improving clinical trial efficiency by addressing common challenges such as high costs, time consumption, and data inaccuracies.

### Leveraging real-world data (RWD) to enhance drug efficacy evaluation

Real-world data is defined as data routinely collected from various sources related to patient health status and/or the delivery of healthcare40. The widespread use of the internet and mobile devices has led to the rapid generation and availability of digital RWD (64).

In this study, we collected real world data from patients through the “Xingyun Family” App, including basic patient information, height, weight, medication usage, medication timing, sleep duration, diet, and living environment. This data provides comprehensive and dynamic information about patients’ health status, reflecting their actual conditions in real world. Compared to traditional clinical trial data, RWD offers more comprehensive and realistic patient information, thereby significantly improving the reliability and practicality of trial results.

Looking forward, it is worthwhile to further utilize mobile apps for large-scale real-world studies. This study conducted as a randomized controlled trial, but has already demonstrated the potential of mobile apps in data collection. Compared to randomized controlled trials, real-world evidence is more generalizable and representative (65). In subsequent research, the App can be developed for real-world studies, allowing users independently record and upload data. For example, users are encouraged to scan the QR (Quick Response) code on the beverage for recording, then to consistently log their intake and sleep data through incentive measures such as point redemption.

This approach enables large-scale data collection, covering diverse regions and populations, providing more comprehensive real-world data. It not only enhances the authenticity and representativeness of the data but also significantly reduces the cost and time. Long-term and large-scale real-world data collection can reveal more detailed and accurate trends and patterns (66), helping to understand the effects of the FBCS on sleep more deeply, and providing strong support for the development of personalized health recommendations and interventions. This data-driven research strategy presents an attractive path for future real-world studies.

## Boundedness

This study has several inevitable limitations. Firstly, patients with psychiatric disorders were excluded to reduce the risk of bias in outcome evaluation. Because this population are with anxiety and depression, which can cause secondary insomnia. This allowed for a more objective assessment of the improvement in sleep quality specifically in patients with primary insomnia. Considering that primary insomnia is characterized by sleep disturbances in the absence of an identifiable underlying psychiatric condition, it was crucial to concentrate exclusively on this group to preserve the study’s validity. However, in modern society, many patients with insomnia also experience anxiety and depression, which may impact the severity and course of their sleep disturbances. Therefore, future studies should consider including patients with comorbid psychiatric conditions to explore the broader effects of FBCS and its potential benefits for this larger group of insomnia patients.

Secondly, the improvement in insomnia with FBCS appears to be gradual. This study only collected data at baseline, on Day 14, and at the endpoint on Day 28, without long-term follow-up. The absence of a longer follow-up period limits the ability to assess the sustainability of the intervention’s effects. Future research should extend the follow-up duration to assess whether the improvements in sleep quality persist over time.

Thirdly, the efficacy assessment and adverse reaction evaluation in this study relied on questionnaires and scales; although the Pittsburgh Sleep Quality Index (PSQI) is considered the gold standard for assessing insomnia, its subjective nature cannot reflect changes in macro-structural sleep characteristics (such as slow-wave sleep duration and REM sleep proportion). Due to constraints imposed by participants engaging in the experiment from home, we attempted to utilize sleep monitoring data collected from participants’ wearable devices, such as smartwatches, to provide insights into sleep structure; however, this data was discarded due to issues with device accuracy. Subsequent studies could incorporate polysomnography (PSG) assessments and measure sleep-related biomarkers (such as salivary melatonin and serum adenosine concentrations) to elucidate FBCS’s dual impact on sleep quality and physiological mechanisms. Additionally, this study did not analyze whether female participants were postmenopausal, which may introduce bias into the statistical results. Menopausal status, a potential confounder due to hormonal fluctuations (e.g., estrogen decline affecting sleep architecture), was not stratified in the analysis. Future studies could incorporate hormonal markers (e.g., follicle-stimulating hormone, luteinizing hormone) to investigate interactions between menopausal status and treatment response, particularly given evidence linking estrogen to adenosine receptor-mediated sleep regulation (53).

## Conclusion

In this study, PSQI results demonstrated that the FBCS significantly improved sleep quality, sleep onset latency, sleep duration, sleep efficiency, sleep disturbances, and daytime function in patients with primary insomnia. Therefore, it can be concluded that FBCS has the potential to significantly enhance sleep conditions in patients with primary insomnia. This study also explored the preliminary integration of clinical trials with Digital Health Tools, offering a new approach to enhancing patient engagement and adherence, warranting further investigation.

## Data Availability

The raw data supporting the conclusions of this article will be made available by the authors, without undue reservation.

## References

[1] Perlis ML Posner D Riemann D Bastien CH Teel J Thase M. Insomnia. Lancet. (2022) 400:1047–60. doi: 10.1016/S0140-6736(22)00879-0, PMID: 36115372

[2] Buysse DJ. Insomnia. JAMA. (2013) 309:706–16. doi: 10.1001/jama.2013.193, PMID: 23423416 PMC3632369

[3] Botchway EN Godfrey C Anderson V Catroppa C. A systematic review of sleep-wake disturbances in childhood traumatic brain injury: relationship with fatigue, depression, and quality of life. J Head Trauma Rehabil. (2019) 34:241–56. doi: 10.1097/HTR.0000000000000446, PMID: 30499928

[4] Brewster GS Riegel B Gehrman PR. Insomnia in the older adult. Sleep Med Clin. (2022) 17:233–9. doi: 10.1016/j.jsmc.2022.03.004, PMID: 35659076

[5] Nassan M Daghlas I Winkelman JW Dashti HS International Suicide Genetics C Saxena R. Genetic evidence for a potential causal relationship between insomnia symptoms and suicidal behavior: a Mendelian randomization study. Neuropsychopharmacology. (2022) 47:1672–9. doi: 10.1038/s41386-022-01319-z, PMID: 35538198 PMC9283512

[6] Alvaro PK Roberts RM Harris JK. A systematic review assessing Bidirectionality between sleep disturbances, anxiety, and depression. Sleep. (2013) 36:1059–68. doi: 10.5665/sleep.2810, PMID: 23814343 PMC3669059

[7] Yu X Mao Y Shergis JL Coyle ME Wu L Chen Y. Effectiveness and safety of oral *Cordyceps sinensis* on stable COPD of GOLD stages 2–3: systematic review and meta-analysis. Evid Based Complement Alternat Med. (2019) 2019:4903671. doi: 10.1155/2019/4903671 31073318 PMC6470429

[8] Shu X Xu D Qu Y Shang X Qiao K Feng C. Efficacy and safety of *Cordyceps sinensis* (*Hirsutella sinensis*, Cs-C-Q80) in chronic bronchitis. Front Pharmacol. (2024) 15:1428216. doi: 10.3389/fphar.2024.1428216, PMID: 39193337 PMC11347402

[9] Wang C Wang J Qi Y. Adjuvant treatment with *Cordyceps sinensis* for lung Cancer: a systematic review and meta-analysis of randomized controlled trials. J Ethnopharmacol. (2024) 327:118044. doi: 10.1016/j.jep.2024.118044, PMID: 38484953

[10] Liu X Zhong F Tang X-l Lian F-l Zhou Q Guo S-m. *Cordyceps sinensis* protects against liver and heart injuries in a rat model of chronic kidney disease: a metabolomic analysis. Acta Pharmacol Sin. (2014) 35:697–706. doi: 10.1038/aps.2013.186, PMID: 24632844 PMC4814030

[11] Wang L Sun H Yang M Xu Y Hou L Yu H. Bidirectional regulatory effects of Cordyceps on arrhythmia: clinical evaluations and network pharmacology. Front Pharmacol. (2022) 13:948173. doi: 10.3389/fphar.2022.948173, PMID: 36059969 PMC9437265

[12] Yang M-L Kuo P-C Hwang T-L Wu T-S. Anti-inflammatory principles from *Cordyceps sinensis*. J Nat Prod. (2011) 74:1996–2000. doi: 10.1021/np100902f, PMID: 21848266

[13] Olatunji OJ Tang J Tola A Auberon F Oluwaniyi O Ouyang Z. The genus Cordyceps: an extensive review of its traditional uses, phytochemistry and pharmacology. Fitoterapia. (2018) 129:293–316. doi: 10.1016/j.fitote.2018.05.010, PMID: 29775778

[14] Dong M Zhao C Huang Y Zheng K Bao G Hu F. Metabolites analysis and new bioactive compounds from the medicine food homology product of Cordyceps Chanhua on artificial media. J Pharm Biomed Anal. (2024) 237:115749. doi: 10.1016/j.jpba.2023.115749, PMID: 37801798

[15] Li Y He L Song H Bao X Niu S Bai J. Cordyceps: alleviating ischemic cardiovascular and cerebrovascular injury-a comprehensive review. J Ethnopharmacol. (2024) 332:118321. doi: 10.1016/j.jep.2024.118321, PMID: 38735418

[16] Pan B-S Lin C-Y Huang B-M. The effect of Cordycepin on steroidogenesis and apoptosis in Ma-10 mouse Leydig tumor cells. Evid Based Complement Alternat Med. (2011) 2011:750468. doi: 10.1155/2011/750468, PMID: 21716681 PMC3118483

[17] Li B Hou Y Zhu M Bao H Nie J Zhang GY. 3’-deoxyadenosine (Cordycepin) produces a rapid and robust antidepressant effect via enhancing prefrontal Ampa receptor signaling pathway. Int J Neuropsychopharmacol. (2016) 19:pyv112. doi: 10.1093/ijnp/pyv112, PMID: 26443809 PMC4851261

[18] Zhou X Gong Z Su Y Lin J Tang K. Cordyceps Fungi: natural products, pharmacological functions and developmental products. J Pharm Pharmacol. (2009) 61:279–91. doi: 10.1211/jpp.61.03.0002, PMID: 19222900

[19] Huang C-W Hong T-W Wang Y-J Chen K-C Pei J-C Chuang T-Y. Ophiocordyceps Formosana improves hyperglycemia and depression-like behavior in an Stz-induced diabetic mouse model. BMC Complement Altern Med. (2016) 16:1–11. doi: 10.1186/s12906-016-1278-7, PMID: 27553852 PMC4995616

[20] Liu YJ Chen J Li X Zhou X Hu YM Chu SF. Research Progress on adenosine in central nervous system diseases. CNS Neurosci Ther. (2019) 25:899–910. doi: 10.1111/cns.13190, PMID: 31334608 PMC6698970

[21] Lazarus M Chen J-F Huang Z-L Urade Y Fredholm BB. Adenosine and sleep. Sleep Wake Neurobiol Pharmacol. Cham: Springer. (2017) 253:359–81. doi: 10.1007/164_2017_36 28646346

[22] Hu Z Lee C-I Shah VK Oh E-H Han J-Y Bae J-R. Cordycepin increases nonrapid eye movement sleep via adenosine receptors in rats. Evid Based Complement Alternat Med. (2013) 2013:840134. doi: 10.1155/2013/840134 23710239 PMC3655593

[23] Stone R. Last stand for the body snatcher of the Himalayas? Science. (2008) 322:1182. doi: 10.1126/science.322.5905.1182, PMID: 19023056

[24] Hopping KA Chignell SM Lambin EF. The demise of Caterpillar fungus in the Himalayan region due to climate change and overharvesting. Proc Natl Acad Sci. (2018) 115:11489–94. doi: 10.1073/pnas.1811591115, PMID: 30348756 PMC6233077

[25] Qin Q-l Zhou G-l Zhang H Meng Q Zhang J-h Wang H-t. Obstacles and approaches in artificial cultivation of Chinese Cordyceps. Mycology. (2018) 9:7–9. doi: 10.1080/21501203.2018.1442132, PMID: 30123655 PMC6059063

[26] Yan M-Q Feng J Liu Y-F Hu D-M Zhang J-S. Functional components from the liquid fermentation of edible and medicinal Fungi and their food applications in China. Food Secur. (2023) 12:2086. doi: 10.3390/foods12102086, PMID: 37238904 PMC10217627

[27] Li S Yang F Tsim KW. Quality control of *Cordyceps sinensis*, a valued traditional Chinese medicine. J Pharm Biomed Anal. (2006) 41:1571–84. doi: 10.1016/j.jpba.2006.01.046, PMID: 16504449

[28] Djurisic S Rath A Gaber S Garattini S Bertele V Ngwabyt SN. Barriers to the conduct of randomised clinical trials within all disease areas. Trials. (2017) 18:360. doi: 10.1186/s13063-017-2099-9, PMID: 28764809 PMC5539637

[29] Mahmud A Zalay O Springer A Arts K Eisenhauer E. Barriers to participation in clinical trials: a physician survey. Curr Oncol. (2018) 25:119–25. doi: 10.3747/co.25.3857, PMID: 29719427 PMC5927782

[30] Alemayehu C Mitchell G Nikles J. Barriers for conducting clinical trials in developing countries-a systematic review. Int J Equity Health. (2018) 17:37. doi: 10.1186/s12939-018-0748-6, PMID: 29566721 PMC5863824

[31] Buysse DJ Reynolds CF III Monk TH Berman SR Kupfer DJ. The Pittsburgh sleep quality index: a new instrument for psychiatric practice and research. Psychiatry Res. (1989) 28:193–213. doi: 10.1016/0165-1781(89)90047-4 2748771

[32] Backhaus J Junghanns K Broocks A Riemann D Hohagen F. Test–retest reliability and validity of the Pittsburgh sleep quality index in primary insomnia. J Psychosom Res. (2002) 53:737–40. doi: 10.1016/s0022-3999(02)00330-6 12217446

[33] Doi Y Minowa M Uchiyama M Okawa M Kim K Shibui K. Psychometric assessment of subjective sleep quality using the Japanese version of the Pittsburgh sleep quality index (Psqi-J) in psychiatric disordered and control subjects. Psychiatry Res. (2000) 97:165–72. doi: 10.1016/s0165-1781(00)00232-8, PMID: 11166088

[34] Galeoto G Scialpi A Grassi ML Berardi A Valente D Tofani M. General sleep disturbance scale: translation, cultural adaptation, and psychometric properties of the Italian version. Cranio. (2021) 39:326–34. doi: 10.1080/08869634.2019.1627067 31181984

[35] Hita-Contreras F Martínez-López E Latorre-Román PA Garrido F Santos MA Martínez-Amat A. Reliability and validity of the Spanish version of the Pittsburgh sleep quality index (Psqi) in patients with fibromyalgia. Rheumatol Int. (2014) 34:929–36. doi: 10.1007/s00296-014-2960-z, PMID: 24509897

[36] Kmetec S Fekonja Z Davey A Mlinar Reljić N Lorber M. Development of a Slovenian version of the Pittsburgh sleep quality index (Psqi-Slo) for use with older adults. Int J Older People Nursing. (2022) 17:e12411. doi: 10.1111/opn.12411, PMID: 34370894 PMC8925995

[37] Sohn SI Kim DH Lee MY Cho YW. The reliability and validity of the Korean version of the Pittsburgh sleep quality index. Sleep and Breathing. (2012) 16:803–12. doi: 10.1007/s11325-011-0579-9, PMID: 21901299

[38] Zhou J Chen X Xiao L Zhou J Feng L Wang G. Efficacy and safety of Cordyceps militaris as an adjuvant to duloxetine in the treatment of insomnia in patients with depression: a 6-week double-blind, randomized, placebo-controlled trial. Front Psych. (2021) 12:754921. doi: 10.3389/fpsyt.2021.754921, PMID: 34858228 PMC8632006

[39] Shen F Wang Q Ullah S Pan Y Zhao M Wang J. Ligilactobacillus Acidipiscis Yj5 modulates the gut microbiota and produces beneficial metabolites to relieve constipation by enhancing the mucosal barrier. Food Funct. (2024) 15:310–25. doi: 10.1039/d3fo03259k, PMID: 38086666

[40] Chen J Wang M Zhang P Li H Qu K Xu R. Cordycepin alleviated metabolic inflammation in Western diet-fed mice by targeting intestinal barrier integrity and intestinal Flora. Pharmacol Res. (2022) 178:106191. doi: 10.1016/j.phrs.2022.106191, PMID: 35346845

[41] Yin X Gou M Xu J Dong B Yin P Masquelin F. Efficacy and safety of acupuncture treatment on primary insomnia: a randomized controlled trial. Sleep Med. (2017) 37:193–200. doi: 10.1016/j.sleep.2017.02.012, PMID: 28899535

[42] Liao Y-H Chao Y-C Sim BY-Q Lin H-M Chen M-T Chen C-Y. Rhodiola/cordyceps-based herbal supplement promotes endurance training-improved body composition but not oxidative stress and metabolic biomarkers: a preliminary randomized controlled study. Nutrients. (2019) 11:2357. doi: 10.3390/nu11102357 31623349 PMC6835767

[43] Chen C-Y Hou C-W Bernard JR Chen C-C Hung T-C Cheng L-L. Rhodiola Crenulata-and Cordyceps Sinensis-based supplement boosts aerobic exercise performance after short-term high altitude training. High Alt Med Biol. (2014) 15:371–9. doi: 10.1089/ham.2013.1114, PMID: 25251930 PMC4174424

[44] Reichert CF Deboer T Landolt HP. Adenosine, caffeine, and sleep-wake regulation: state of the science and perspectives. J Sleep Res. (2022) 31:e13597. doi: 10.1111/jsr.13597, PMID: 35575450 PMC9541543

[45] Zhang X Liu Q Zhou W Li P Alolga RN Qi L-W. A comparative proteomic characterization and nutritional assessment of naturally-and artificially-cultivated *Cordyceps sinensis*. J Proteome. (2018) 181:24–35. doi: 10.1016/j.jprot.2018.03.029, PMID: 29609095

[46] Kim YO Kim HJ Abu-Taweel GM Oh J Sung G-H. Neuroprotective and therapeutic effect of Cordyceps Militaris on ischemia-induced neuronal death and cognitive impairments. Saudi J Biol Sci. (2019) 26:1352–7. doi: 10.1016/j.sjbs.2018.08.011, PMID: 31762595 PMC6864366

[47] Huang S-Y Su Z-Y Han Y-Y Liu L Shang Y-J Mai Z-F. Cordycepin improved the cognitive function through regulating adenosine A2a receptors in Mptp induced Parkinson's disease mice model. Phytomedicine. (2023) 110:154649. doi: 10.1016/j.phymed.2023.154649, PMID: 36634379

[48] Lazarus M Oishi Y Bjorness TE Greene RW. Gating and the need for sleep: dissociable effects of adenosine a (1) and a (2a) receptors. Front Neurosci. (2019) 13:740. doi: 10.3389/fnins.2019.00740, PMID: 31379490 PMC6650574

[49] Chen X Zhang X-L Wang C-M Feng L Wang G. Cordyceps Sinensis combined with duloxetine improves sleep symptoms in patients with depression: a randomized, double-blind, placebo-controlled study. Asia Pacific J Clinical Trials Nervous System Diseases. (2018) 3:136–45. doi: 10.4103/2542-3932.245217, PMID: 39968893

[50] Kaptchuk TJ Miller FG. Placebo effects in medicine. N Engl J Med. (2015) 373:8–9. doi: 10.1056/NEJMp1504023, PMID: 26132938

[51] Finniss DG Kaptchuk TJ Miller F Benedetti F. Biological, clinical, and ethical advances of placebo effects. Lancet. (2010) 375:686–95. doi: 10.1016/S0140-6736(09)61706-2, PMID: 20171404 PMC2832199

[52] Ho YT Tsai YC Kuo TBJ Yang CCH. Effects of *Lactobacillus plantarum* Ps128 on depressive symptoms and sleep quality in self-reported insomniacs: a randomized, double-blind, placebo-controlled pilot trial. Nutrients. (2021) 13:2820. doi: 10.3390/nu13082820, PMID: 34444980 PMC8402034

[53] de Zambotti M Colrain IM Baker FC. Interaction between reproductive hormones and physiological sleep in women. J Clin Endocrinol Metab. (2015) 100:1426–33. doi: 10.1210/jc.2014-3892, PMID: 25642589 PMC4399298

[54] Xu H Zhang C Qian Y Zou J Li X Liu Y. Efficacy of melatonin for sleep disturbance in middle-aged primary insomnia: a double-blind, randomised clinical trial. Sleep Med. (2020) 76:113–9. doi: 10.1016/j.sleep.2020.10.018, PMID: 33157425

[55] Vermeire E Hearnshaw H Van Royen P Denekens J. Patient adherence to treatment: three decades of research. A comprehensive review. J Clin Pharm Ther. (2001) 26:331–42. doi: 10.1046/j.1365-2710.2001.00363.x, PMID: 11679023

[56] van Dulmen S Sluijs E van Dijk L de Ridder D Heerdink R Bensing J. Patient adherence to medical treatment: a review of reviews. BMC Health Serv Res. (2007) 7:55. doi: 10.1186/1472-6963-7-55, PMID: 17439645 PMC1955829

[57] Burnier M. Physician and patient adherence in hypertension trials: a point of view on an important issue to resolve. Expert Rev Pharmacoecon Outcomes Res. (2024) 24:749–58. doi: 10.1080/14737167.2024.2363401, PMID: 38836304

[58] Kearney A Rosala-Hallas A Bacon N Daykin A Shaw ARG Lane AJ. Reducing attrition within clinical trials: the communication of retention and withdrawal within patient information leaflets. PLoS One. (2018) 13:e0204886. doi: 10.1371/journal.pone.0204886, PMID: 30379822 PMC6209179

[59] Tate A Smallwood C. Comparing the efficiency of paper-based and electronic data capture during face-to-face interviews. PLoS One. (2021) 16:e0247570. doi: 10.1371/journal.pone.0247570, PMID: 33684116 PMC7939350

[60] Pavlović I Kern T Miklavčič D. Comparison of paper-based and electronic data collection process in clinical trials: costs simulation study. Contemp Clin Trials. (2009) 30:300–16. doi: 10.1016/j.cct.2009.03.008, PMID: 19345286

[61] Armitage LC Kassavou A Sutton S. Do Mobile device apps designed to support medication adherence demonstrate efficacy? A systematic review of randomised controlled trials, with Meta-analysis. BMJ Open. (2020) 10:e032045. doi: 10.1136/bmjopen-2019-032045, PMID: 32005778 PMC7045248

[62] Dzavakwa NV Kranzer K Khan P Mackworth-Young CRS Mujuru HA Ferrand RA. Electronic monitoring device informed interventions for treatment adherence and clinical outcomes in children and adolescents: a systematic review. Int J Nurs Stud. (2024) 160:104903. doi: 10.1016/j.ijnurstu.2024.104903, PMID: 39303643

[63] Ryu H Piao M Kim H Yang W Kim KH. Development of a mobile application for smart clinical trial subject data collection and management. Appl Sci. (2022) 12:3343. doi: 10.3390/app12073343

[64] Liu F Panagiotakos D. Real-world data: a brief review of the methods, applications, challenges and opportunities. BMC Med Res Methodol. (2022) 22:287. doi: 10.1186/s12874-022-01768-6, PMID: 36335315 PMC9636688

[65] Silva P Janjan N Ramos KS Udeani G Zhong L Ory MG. External control arms: COVID-19 reveals the merits of using real world evidence in real-time for clinical and public health investigations. Front Med. (2023) 10:1198088. doi: 10.3389/fmed.2023.1198088, PMID: 37484840 PMC10359981

[66] Roche N Reddel H Martin R Brusselle G Papi A Thomas M. Quality standards for real-world research. Focus on observational database studies of comparative effectiveness. Ann Am Thorac Soc. (2014) 11:S99–S104. doi: 10.1513/AnnalsATS.201309-300RM, PMID: 24559028

